# Supplementary material for: DMRforPairs: identifying Differentially Methylated Regions between unique samples using array based methylation profiles
Source: BMC Bioinformatics. 2014 May 15;15:141. doi: 10.1186/1471-2105-15-141 (PMC4046028; doi:10.1186/1471-2105-15-141)
Supplement: Additional file 2 — DMRforPairs output for the comparison of A431-MCF7 and NA17018-NA17105. Please start from the HTML files in each folder. Available via the BMC Bioinformatics website. [file 1471-2105-15-141-S2.zip › 1394847754114233_MOESM2_ESM/A431_MCF7/figures/35969.pdf]

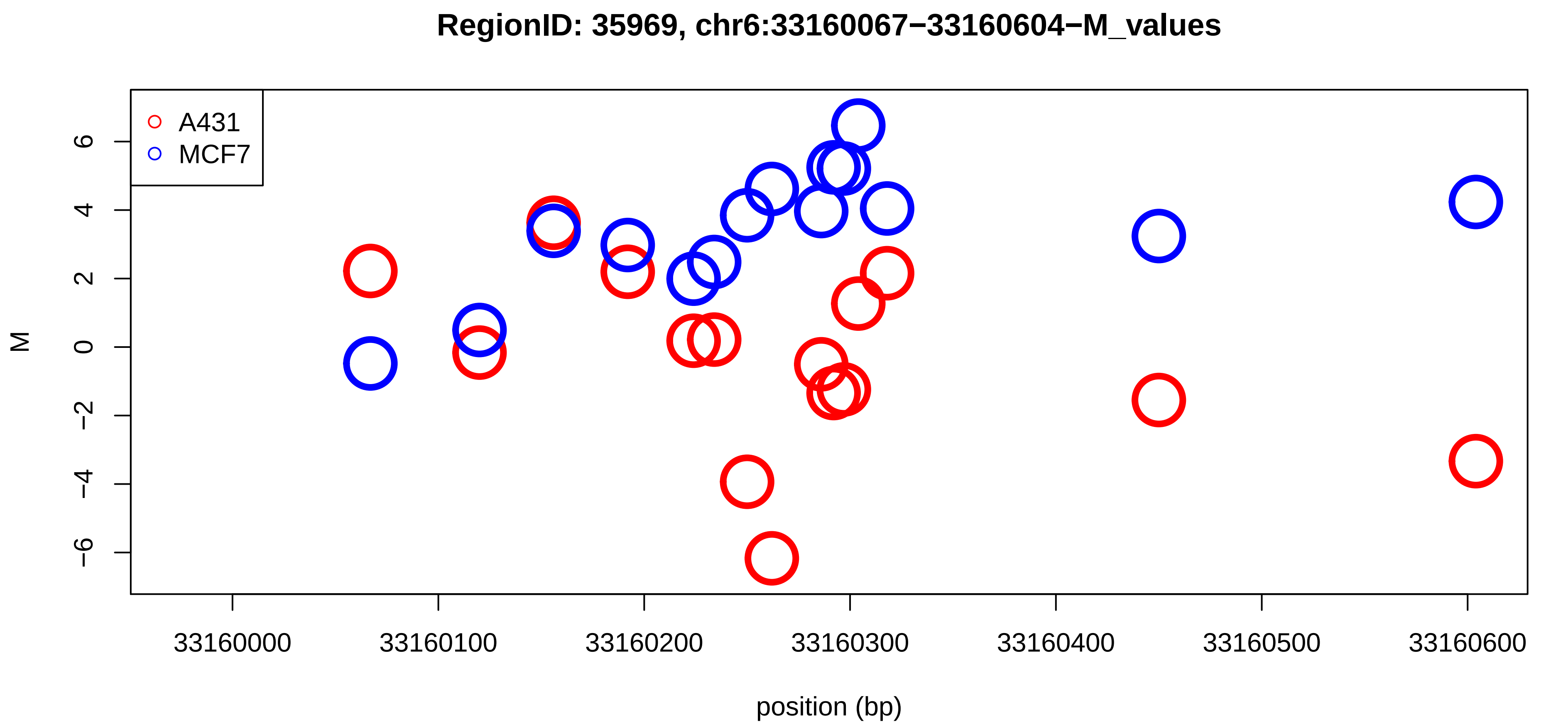
RegionID: 35969, chr6:33160067−33160604−M_values
A431
MCF7
M
6
4
2
0
−2
−4
−6
33160000
33160100
33160200
33160300
33160400
33160500
33160600
position (bp)

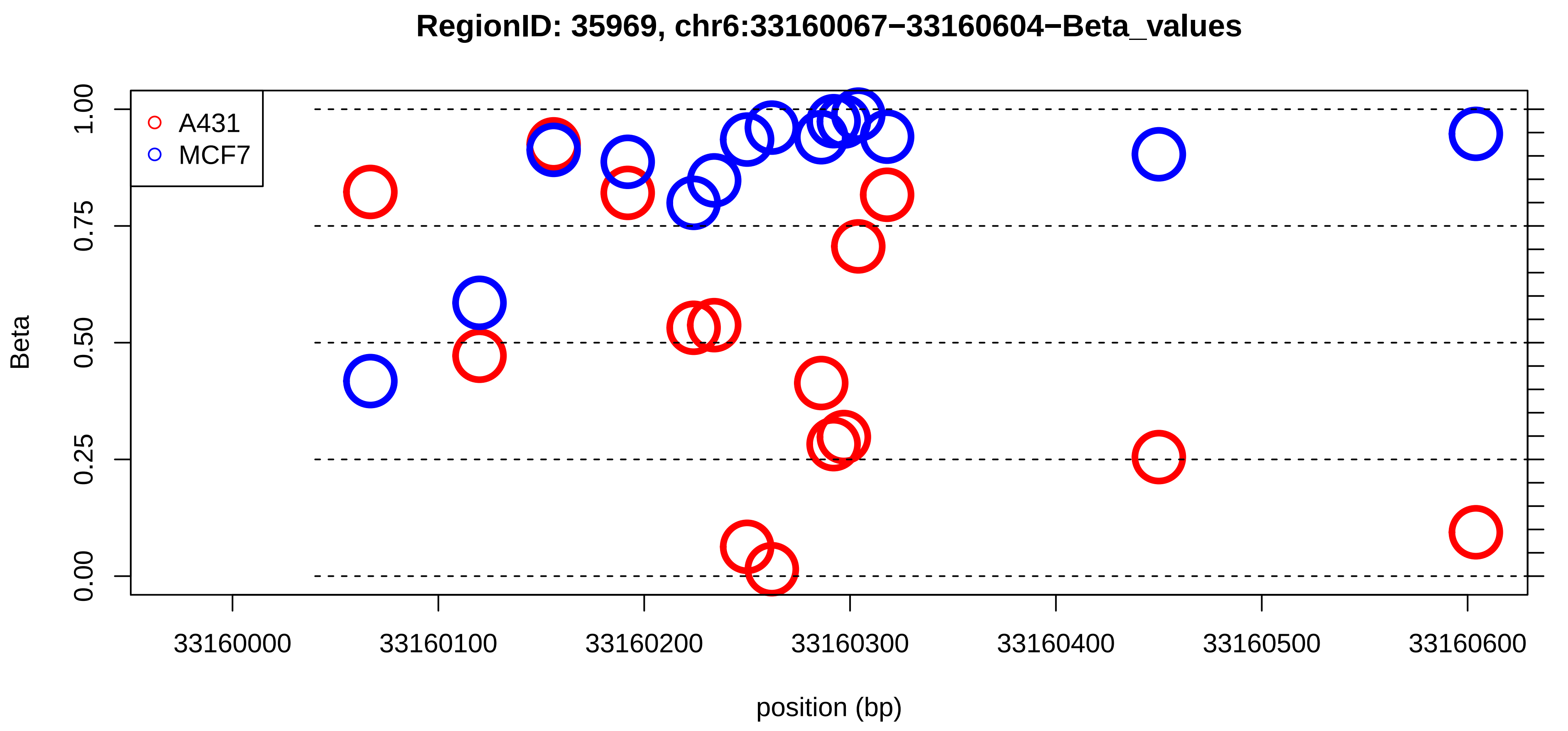
RegionID: 35969, chr6:33160067−33160604−Beta_values
A431
MCF7
Beta
1.00
0.75
0.50
0.25
0.00
33160000
33160100
33160200
33160300
33160400
33160500
33160600
position (bp)

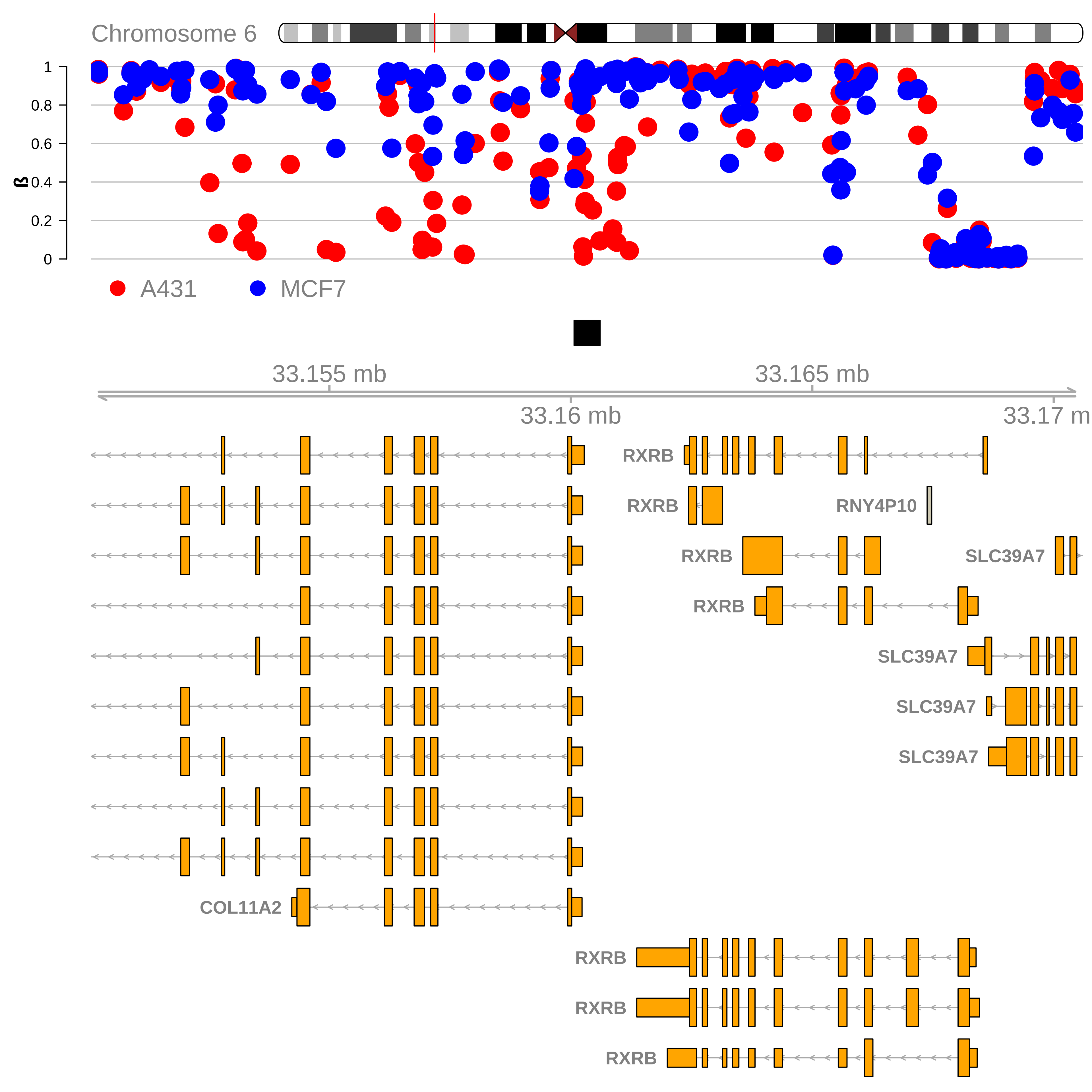

Chromosome 6
1
0.8
0.6
ß 0.4
0.2
0
A431
MCF7
33.155 mb
33.165 mb
33.16 mb
33.17 m
RXRB
RXRB
RNY4P10
RXRB
SLC39A7
RXRB
SLC39A7
SLC39A7
SLC39A7
COL11A2
RXRB
RXRB
RXRB
